# Ultimate suppression of thermal transport in amorphous silicon nitride by phononic nanostructure

**DOI:** 10.1126/sciadv.abc0075

**Published:** 2020-09-25

**Authors:** Naoki Tambo, Yuxuan Liao, Chun Zhou, Elizabeth Michiko Ashley, Kouhei Takahashi, Paul F. Nealey, Yasuyuki Naito, Junichiro Shiomi

**Affiliations:** 1 Technology Division, Panasonic Corporation, Kyoto, Japan.; 2 Department of Mechanical Engineering, The University of Tokyo, Tokyo, Japan.; 3 Pritzker School of Molecular Engineering, University of Chicago, Chicago, IL, USA.; 4 Materials Science Division, Argonne National Laboratory, Argonne, IL, USA.

## Abstract

Engineering the thermal conductivity of amorphous materials is highly essential for the thermal management of future electronic devices. Here, we demonstrate the impact of ultrafine nanostructuring on the thermal conductivity reduction of amorphous silicon nitride (a-Si_3_N_4_) thin films, in which the thermal transport is inherently impeded by the atomic disorders. Ultrafine nanostructuring with feature sizes below 20 nm allows us to fully suppress contribution of the propagating vibrational modes (propagons), leaving only the diffusive vibrational modes (diffusons) to contribute to thermal transport in a-Si_3_N_4_. A combination of the phonon-gas kinetics model and the Allen-Feldmann theory reproduced the measured results without any fitting parameters. The thermal conductivity reduction was explained as extremely strong diffusive boundary scattering of both propagons and diffusons. These findings give rise to substantial tunability of thermal conductivity of amorphous materials, which enables us to provide better thermal solutions in microelectronic devices.

## Teaser

Nanostructuring of amorphous solids can fully suppress the propagating lattice vibrations, leaving only diffusive heat carrier.

## INTRODUCTION

Amorphous materials play an essential role in modern semiconductor devices (*1*), such as photoelectric conversion layers for solar cells, phase change memory, thin-film transistors for displays, microelectromechanical systems, thermoelectric devices, and gate dielectrics and interlayer dielectrics for complementary metal-oxide semiconductor technology. The behavior of these devices depends strongly on the operation temperatures, which makes thermal management an important issue that needs to be tackled to ensure their performance and reliability. Therefore, understanding the thermal transport properties of amorphous solids is extremely important to optimize the thermal design of microelectronic devices. In particular, as the size of the device components scales down to the nanometer order, this aspect becomes more important given that unusual thermal transport properties have been identified in crystalline solids with nanometer feature sizes.

However, heat transport in amorphous solids is much more complicated than that of the crystalline solids despite the fact that heat is similarly carried by atomic vibrations. Heat conduction in crystalline solids with complete periodicity is well understood in terms of mode-dependent phonon transport properties. The thermal conductivity κ of crystalline solids obtained by computational work has thus shown good agreement with measurements (*2*, *3*). Theoretical interpretation is performed on the basis of the phonon Boltzmann transport equation, which requires phonons to have well-defined group velocities. However, for the amorphous solids, only a small portion of vibrational modes with low frequencies are considered to have well-defined group velocity. Seminal work by Allen and Feldman described that the vibrational modes in amorphous materials can be classified into three categories, namely, propagons, diffusons, and locons (*4*–*6*). The nonlocalized vibration modes at low frequencies that exhibit wave-like features are called propagons. On the other hand, a large amount of density of states is occupied by the nonlocalized vibrational modes at higher frequencies called diffusons, which conduct heat in a rather diffusive manner. Allen and Feldman expressed κ as a function of mode “diffusivity” to describe diffuson transport (AF theory) (*4*, *5*). Propagons and diffusons are nonlocalized modes contributing to the heat transport, whereas locons are localized vibration mode, which does not contribute to κ.

Following the AF theory, several studies have focused on the properties of vibration modes in amorphous solids (*1*, *7*), for example, the definition of threshold frequency between the three vibration modes, the contribution of each vibration modes to the total κ of solids, and their size effects. Several criteria have been proposed to classify propagons and diffusons, such as by the vibrational mode density of states (*6*, *8*), the eigenvector periodicity (*9*), and the dynamical structure factor intensity (*10*). Theoretical studies showed that propagon contributes ~40% of the total κ of amorphous silicon and that their mean free path (MFP) extends up to 1 μm (*8*, *11*). Experimental studies have revealed that both the cross-plane κ (*12*, *13*) and the in-plane κ of amorphous films depend on the feature size of the material (*14*–*16*). These experimental studies supported that propagons have relatively long MFPs up to 1 μm and contribute up to 50% of total κ. It is also noteworthy that the propagons showed a ballistic transport feature in short distances (*16*), which is similar to phonons in crystalline solids.

The previous studies thus indicate that the thermal transport properties of amorphous solids can be controlled by fine nanostructuring in analogy with those of crystalline materials, which has been demonstrated in the form of nanowires (*17*, *18*), superlattices (*19*, *20*), and holey phononic crystals (PnCs) (*21*–*23*). Similar approaches used in “phonon engineering” (*24*) may be effective in the manipulation of thermal transport properties of amorphous materials as well. However, we still lack a quantitative understanding of nanoscale thermal transport of amorphous materials. Systematic measurements of samples with a wide variety of feature sizes, together with supportive theoretical simulation mimicking the approaches in phonon engineering, will be indispensable to fully leverage the phonon engineering techniques developed in crystalline materials.

In this study, we experimentally and theoretically investigated the thermal transport properties of amorphous silicon nitride (a-Si_3_N_4_) with ultrafine phononic nanostructures. The samples examined here are suspended a-Si_3_N_4_ thin films with holey PnC structures, which have a periodic two-dimensional array of through-holes aligned at various pitch sizes ranging widely from several tens of nanometers to micrometer order. The wide variety of sample feature sizes investigated here allowed us to clearly identify the effect of nanostructuring on the thermal transport properties of a-Si_3_N_4_. The effect of boundary scattering on propagons and diffusons was quantitatively analyzed by a modified simulation model based on a Monte Carlo ray tracing (MCRT) (*25*) method, which was developed previously for describing the phonon transport mechanism in crystalline solids. We found that the theoretical calculation reproduces the measured κ of a-Si_3_N_4_ well throughout the samples investigated here. The current result not only deepens our understanding of nanoscale thermal transport of amorphous solids but also demonstrates that the approaches developed in phonon engineering are highly applicable to propagon and diffuson engineering as well.

## RESULTS AND DISCUSSION

**Fig. 1:**
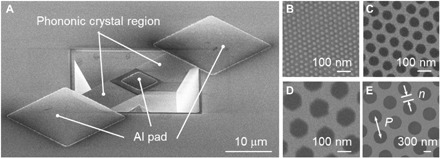
Measured samples and patterning dimension definition. Scanning electron microscopy (SEM) images of amorphous Si_3_N_4_ PnCs. (**A**) Overall image of the sample. Al pads are deposited on the center and the edges of the suspended bridge for the thermal conductivity measurement. High-magnification SEM image of the aligned through-holes creating PnCs for pitch sizes of (**B**) 36 nm, (**C**) 100 nm, (**D**) 200 nm, and (**E**) 800 nm. The definition of pitch size *P* and minimum neck width *n* is illustrated in (E).

Figure 1 summarizes the scanning electron microscopy (SEM) images of our samples. a-Si_3_N_4_ thin films were deposited on Si substrates by using a low-pressure chemical vapor deposition (LPCVD) method. Periodic through-holes with pitch *P* sizes from 60 to 1600 nm were patterned on the a-Si_3_N_4_ films by electron beam lithography (Fig. 1, C to E), whereas those with *P* of 36 nm were fabricated by directed self-assembly lithography (Fig. 1B) (*26*). The holes of the PnC structures are aligned in a triangular pattern (Fig. 1). Here, we define the minimum neck width *n* of PnC structures as *n* = *P* − *D*, where *D* is the diameter of the holes. Note that *n* ranges from 11 to 670 nm. Detailed geometries of the PnC structures are shown in Table 1. The a-Si_3_N_4_ films are partially suspended from the base substrate so as to avoid the influence of the substrate on the measured κ (Fig. 1A). The length, width, and thickness of the suspended bridge are 30 μm, 10 μm, and 70 nm, respectively. Al pads with thickness of 130 nm are deposited on the center and the edge of the suspended bridge structure for the thermal transport measurements. Detailed information on experimental fabrication processes is discussed in the "Fabrication of PnCs” section and in our recent report (*27*).

**Table 1:** Lists of amorphous Si_3_N_4_ PnC structures with a thickness of 70 nm.

**Pitch (nm)**	**Porosity**	**Diameter (nm)**	**Neck width** **(nm)**
36	0.44	25	11
60	0.30	35	25
60	0.36	38	22
60	0.39	39	21
100	0.44	70	30
100	0.50	74	26
100	0.53	76	24
100	0.59	81	19
200	0.39	132	68
200	0.47	144	56
200	0.54	154	46
200	0.58	160	40
200	0.69	175	25
400	0.39	262	138
400	0.46	285	115
400	0.57	317	83
800	0.36	506	294
800	0.44	557	243
800	0.50	594	206
1600	0.31	935	665
1600	0.36	1013	587
1600	0.40	1063	537

The thermal transport property of a-Si_3_N_4_ PnCs was measured by a time-domain thermoreflectance (TDTR) method (*23*), which is a well-established method based on a pump-probe optical measurement. In our TDTR setup, a continuous-wave laser at a wavelength of 785 nm was used as the probe beam, whereas a quasi-continuous wave laser with a pulse duration of 4 μs at a wavelength of 852 nm was used as the pump beam. Both the pump beam and probe beam were focused on the Al pad deposited on the center of the suspended bridge structure. The probe beam was used to monitor the temperature-dependent reflectance change of the Al pad, while the pump beam was used to heat the Al pad. All measurements were carried out at 300 K. The peak power of the pump beam was set at 1 mW, while that of the probe beam was set at 30 μW. We confirmed that the temperature rise due to the laser irradiation had negligible influence on the measured κ, which was checked by carrying out the measurement at multiple laser power. The samples were placed under vacuum at a pressure of 5 × 10^−4^ Pa to exclude the influence of convection on the thermal relaxation behavior of the Al pad. The standard error was smaller than ±3% for each sample in our measurement. The TDTR signal exhibits a sharp rise in intensity, which is followed by an exponential decay. The material thermal conductivity κ_mat_ of a-Si_3_N_4_ PnCs, which does not include the classical geometric effect of the pores, can be obtained by fitting the experimental TDTR signal with that of the TDTR signal simulated by a finite element method (FEM). The influence of pores on the effective thermal conductivity κ_T_ of the a-Si_3_N_4_ films can be calculated accurately using FEM analysis. We used ANSYS software for the FEM analysis. More detailed information is summarized in the “Measurement of thermal conductivity” section.

**Fig. 2:**
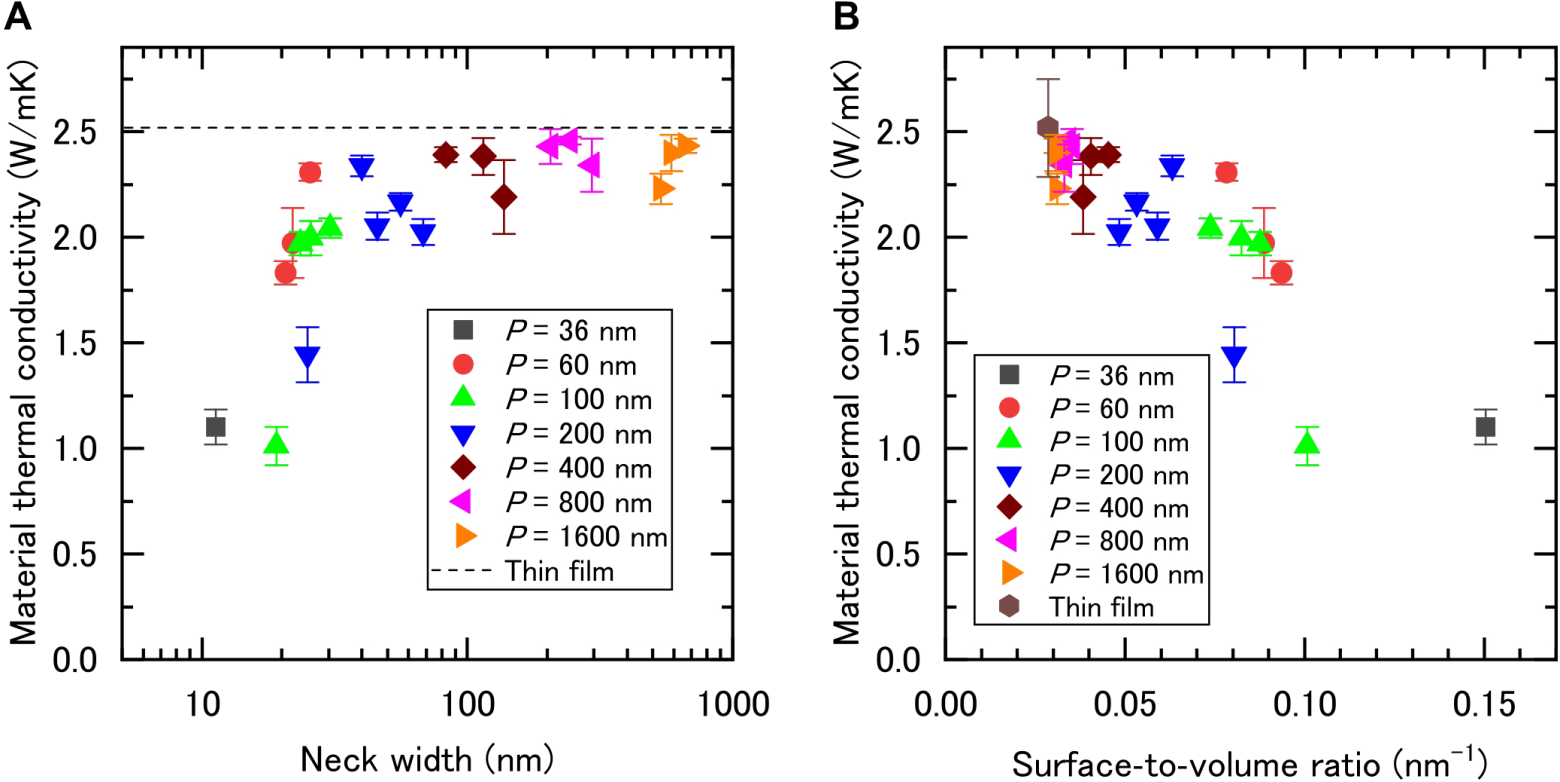
Material thermal conductivity of amorphous Si_3_N_4_ PnCs. Material thermal conductivity of amorphous Si_3_N_4_ thin films plotted as a function of (**A**) the minimum neck width and (**B**) the surface-to-volume (S/V) ratio. Plot legends denote the pitch size of PnCs. The black dashed line in (A) represents the thermal conductivity of bare amorphous Si_3_N_4_ thin films.

The measured κ_mat_ of the bare thin films and PnCs as a function of *n* are shown in Fig. 2A. κ_mat_ of the 70-nm-thick bare films was 2.5 ± 0.2 W/mK, which was consistent with the previous study measured by the 3ω method (*28*). As shown in Fig. 2A, κ_mat_ of PnCs shows a decreasing trend with decreasing *n*. In particular, κ_mat_ shows a steep decrease in its magnitude when *n* is reduced below 20 nm, where it exhibits a substantially low value of ~1 W/mK. This result clearly demonstrates that κ_mat_ of the amorphous solids can be manipulated by the PnC nanostructures.

According to the previous studies, the propagating vibrational modes with relatively long MFP should dominate the magnitude of κ_mat_. As is the case with phonons in crystalline solids, we assume that transport of these propagating modes in a-Si_3_N_4_ is similarly disturbed by boundary scattering. To achieve further insight into the effect of boundary scattering, we show in Fig. 2B the measured κ_mat_ of a-Si_3_N_4_ PnCs as a function of surface-to-volume (S/V) ratio. We see that κ_mat_ indeed decreases with increasing the S/V ratio. This is consistent with the feature reported for crystalline Si (c-Si) PnCs (*29*) where the reduction in κ was attributed to enhanced boundary scattering of phonons due to an increase in the S/V ratio. The result indicates that the reduction in κ_mat_ of the present a-Si_3_N_4_ PnCs is indeed related to enhanced boundary scattering of propagons.

Another remarkable feature observed in Fig. 2B is that κ_mat_ converges to a certain value (~1 W/mK) after a monotonic decrease when the S/V ratio is increased above 0.1 nm^−1^, which indicates that propagons are fully suppressed, leaving only diffusons to dominate thermal transport. Here, we thus call this converged κ value of ~1 W/mK as the “diffusive limit,” which represents the κ contribution of diffusons in bulk a-Si_3_N_4_.

Similar reduction trend of κ in terms of S/V ratio was also reported in c-Si PnCs and nanowires. However, their κ reduction rate is much larger than that of a-Si_3_N_4_ PnCs. The reason is that the MFP of phonons that govern κ at room temperature in c-Si is distributed in a range of 100 nm to 10 μm, whereas that of heat carriers in a-Si_3_N_4_ is mainly distributed in a range of 1 to 100 nm (fig. S1). Therefore, phonons in c-Si are much more sensitive to boundary scatterings. Moreover, as the S/V ratio increases, localized phonons become more important to thermal transport and lastly make κ saturate, as indicated in the work of Chen *et al*. (*30*) for nanowires. Although the localization of propagons may also take place in a-Si_3_N_4_, our theoretical calculation implies that the saturation of κ observed here is mainly related to the suppression of propagon transport by boundary scattering. As we show in the following paragraphs, κ contributed by diffusons in bulk a-Si_3_N_4_ was calculated to be 1.1 W/mK, which is consistent with the converged κ value shown in Fig. 2 (A and B). The good agreement supports that the heat is mainly carried by diffusons in the a-Si_3_N_4_ PnCs that exhibit S/V ratio larger than 0.1 nm^−1^.

Propagons are phonon-like propagating vibrational modes, and thus, we assume that the thermal conductivity contribution from propagons (κ_P_) follow the phonon gas model as

$$\kappa_{P}=\frac{1}{3V}\sum_{i,\omega_{i}<\omega_{t}}C(\omega_{i})\text{DOS}(\omega_{i}){v_{s}}^{2}\;\tau (\omega_{i})$$ (1)

where *V* is the system volume, ω_t_ is the transition frequency of propagons and diffusons, which is determined as 4 THz (details are summarized in section S1), *v*_s_ is the appropriate sound speed, τ(ω) is the frequency-dependent relaxation time, and DOS (ω) is the vibrational density of states. *C*(ω) is the mode-dependent specific heat capacity described as

$$C(\omega )=k_{B}{\left[\frac{\frac{\hbar \omega }{2k_{B}T}}{\text{sinh}(\frac{\hbar \omega }{2k_{B}T})}\right]}^{2}$$ (2)

where *k*_B_ is the Boltzmann constant, ℏ is the reduced Planck constant, and *T* is temperature. Here, we focus on room temperature, i.e., 300 K.

The dispersion relation of propagons is expected to be linear, similar to that of sound. Thus, their DOS is assumed to obey the Debye approximation. DOS is then described as

$$\text{DOS}(\omega )=\frac{3V\omega^{2}}{2\pi^{2}v_{s}^{3}}$$ (3)

On the other hand, the thermal conductivity contribution from diffusions (κ_D_) can be described by the AF theory as

$$\kappa_{D}=\frac{1}{V}\Sigma_{i,\omega_{i}>\omega_{t}}C(\omega_{i})D(\omega_{i})$$ (4)

where ω*_i_* is the frequency of the *i*-th diffuson mode, and *D*(*ω_i_*) is the diffuson diffusivity, which can be calculated as

$$D(\omega_{i})=\frac{\pi V^{2}}{\hbar^{2}\omega_{i}^{2}}\Sigma_{j\neq i}{S_{\mathrm{ij}}}^{2}\delta (\omega_{i}-\omega_{j})$$ (5)

where δ is the delta function broadened into Lorentzian, and *S_ij_* is the heat current operator as a function of frequencies and eigenvectors, which can be calculated from the harmonic lattice dynamics theory.

The relaxation time of each vibration mode can be obtained by performing the normal mode decomposition (NMD) analysis on the phase space trajectories obtained by equilibrium molecular dynamics (MD) simulation. Since propagons are similar to phonons with low frequencies, the relaxation time of propagons can be modeled with the form similar to the following Klemens model, which is widely used and has been validated for low-frequency phonons in various kinds of materials. This is described as

$$\tau (\omega )=B\omega^{-2}$$ (6)

where *B* is a constant coefficient that incorporates the effect of scatterings and temperatures. The magnitude of *B* can be evaluated by fitting the relaxation time of propagons from NMD calculations.

To quantitatively evaluate boundary scattering of propagons and diffusons in amorphous PnCs, we need to define their MFP. For phonon-like propagons, the MFP is defined by the appropriate sound speed and relaxation time as

$$\Lambda (\omega )=v_{s}\tau (\omega )$$ (7)

For diffusons, the MFP is defined by their diffusive length, which can be obtained by comparing Eqs. 1 and 4

$$D(\omega )=\frac{1}{3}v_{D}^{2}\tau (\omega )=\frac{1}{3}v_{D}\Lambda (\omega )$$ (8)

$$\Lambda (\omega )=\sqrt{3D(\omega )\tau (\omega )}$$ (9)

where *v*_D_ is the diffusive velocity of diffusons, and τ(ω) is the frequency-dependent relaxation time of diffusons obtained by the NMD calculations.

Using Eqs. 8 and 9, the thermal conductivity of bulk amorphous solids κ_T_ (= κ_P_ + κ_D_) can be expressed in the form similar to that of the phonon gas model as

$$\kappa_{T}=\frac{1}{3V}\Sigma_{i}C(\omega_{i})\text{DOS}(\omega_{i})v_{s,D}\;(\omega_{i})\Lambda (\omega_{i})$$ (10)

where, *v*_s_,_D_ represents *v*_s_ for propagons and *v*_D_ for diffusons. Note that, here, we defined the effective MFP of propagons and diffusons in a unified way; however, we still can identify their contributions to κ_T_ by their unique features such as the frequency-dependent DOS and the effective dispersion relations of propagons, as shown in section S2.

The calculation of the bulk properties of a-Si_3_N_4_ was similar to the works of Larkin and McGaughey (*8*) (detailed information is summarized in the “Calculation of bulk thermal properties of silicon nitrides” section and section S2). On the basis of this model, we have calculated the room temperature κ_T_ of bulk a-Si_3_N_4_. The obtained value was 2.9 W/mK, which is consistent with the measured κ reported by Sultan *et al.* (*31*), Zink and Hellman (*32*), and Ftouni *et al.* (*28*). The calculated κ_P_ and κ_D_ were identified as 1.8 and 1.1 W/mK, respectively. This indicates that propagons with long MFP can contribute to a greater part (62%) of the κ_T_ of bulk a-Si_3_N_4_, which is consistent with the work reported by Sultan *et al.* (*14*) (50%).

Now that we confirmed the validity of our model in bulk a-Si_3_N_4_, we move on to discussing the thermal transport property of a-Si_3_N_4_ thin films and PnCs. To incorporate the effect of boundary scattering in the bulk model, we used the MCRT method, which has been a powerful tool to quantify the boundary scattering of phonons in crystalline solids (*25*). The MCRT method provides us the effective MFP of propagons and diffusons of thin films and PnCs, which, in turn, provides us the magnitude of κ_T_ of the a-Si_3_N_4_ thin films and PnCs.

The calculated κ of the 70-nm-thick films was 2.3 W/mK, which was consistent with our measured value of 2.5 ± 0.2 W/mK. Because of boundary scattering of propagons, 21% reduction in κ was identified with respect to that of bulk a-Si_3_N_4_. In contrast, diffusons were not affected by the boundary of the 70-nm-thick films because of their very short MFP, which range from several angstroms to several nanometers. As mentioned above, κ_D_ of the bulk a-Si_3_N_4_ was calculated to be 1.1 W/mK. This means that κ_D_ of the 70-nm-thick a-Si_3_N_4_ films is 1.1 W/mK as well. One of the remarkable aspects here is that the calculated κ_D_ of thin films agrees well with the measured diffusive limit (1.01 ± 0.09 W/mK). This result supports our discussion that propagons can be drastically suppressed by the PnC structures, leaving only the diffusons to contribute to the thermal transport in a-Si_3_N_4_ PnCs when *n* is reduced below 20 nm.

**Fig. 3:**
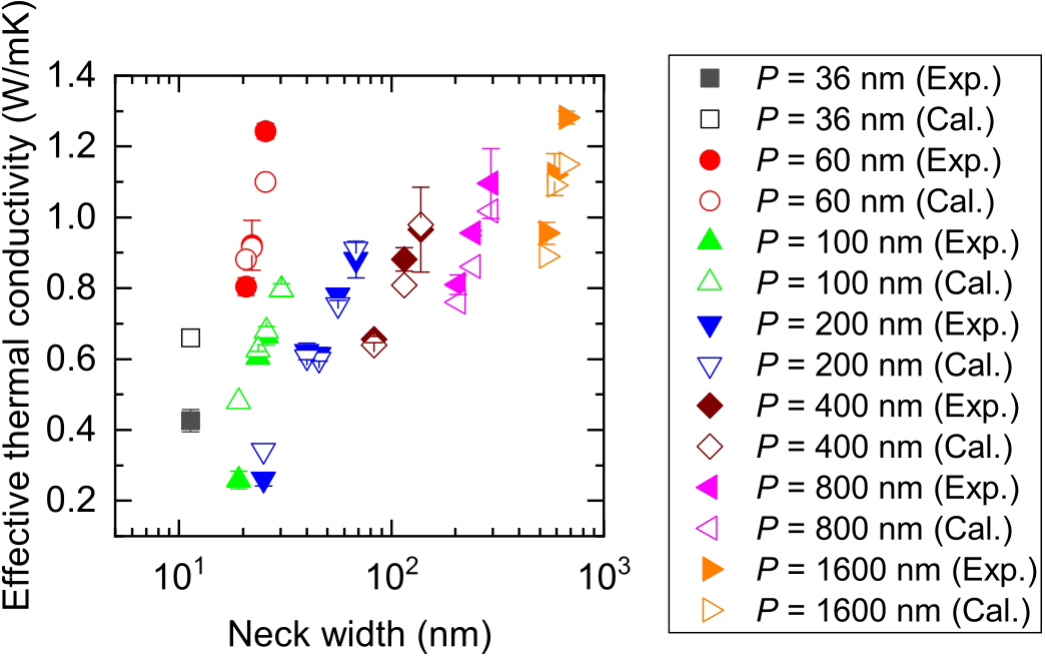
Effective thermal conductivity of amorphous Si_3_N_4_ PnCs. Comparison between the measured and the simulated effective thermal conductivity of amorphous Si_3_N_4_ PnCs plotted as a function of the neck width. Plot legends denote the pitch size of PnCs, where Exp. and Cal. denote Experiment and Calculation.

In Fig. 3, we compare our experimental results and the simulated results for various kinds of a-Si_3_N_4_ PnCs. Here, the measured κ_mat_ was converted into κ_T_ using the Maxwell-Garnett model expressed by

$$\kappa_{T}=\frac{1-\varphi }{1+\varphi }\kappa_{\text{mat}}$$ (11)

where φ is the porosity of the material. The validity of the Maxwell-Garnett model for our samples was confirmed using the steady-state thermal analysis module of ANSYS software. We find that κ_T_ of these PnCs is substantially reduced with respect to that of the thin films. The lowest κ_T_ obtained here was 0.26 ± 0.03 W/mK, which is comparable with typical plastic materials such as polyethylene (κ = 0.367 W/mK) (*33*). We can also see that the calculation agrees well with the measurement. This indicates that the model developed here originally based on phonon simulation can be useful to reproduce the thermal transport properties of amorphous PnCs. For most of the a-Si_3_N_4_ PnCs, the difference between the calculated values and the experimental values is within 10%. The only exception was observed in samples with *n* = 19 nm (*P* = 100 nm and *D* = 81 nm), which showed an acceptable but relatively large 35% difference. A possible reason for the discrepancy can be attributed to the difficulty in accurately determining *n* at this small size scale.

Coherent phonon transport is generally highlighted when we discuss the thermal transport properties of periodic nanostructures. Evidence of the band folding effect or impact of periodicity on thermal transport, which all indicate the presence of coherent phonon transport, has been observed in various crystalline nanomaterials. Band folding has also been observed in amorphous superlattices by Koblinger *et al.* (*34*). However, the presence of coherent transport of propagons in amorphous phononic materials still remains as an open question. Here, our theoretical calculations showed that the measured κ of a-Si_3_N_4_ phononic materials can be reproduced by the particle-based Boltzmann transport equation. This indicates that coherent thermal transport is not substantial in a-Si_3_N_4_ PnCs at 300 K even when *P* is reduced down to 36 nm. However, we believe that coherent transport of propagons can become important at low temperatures as observed in crystalline PnCs (*35*), where low-frequency propagons dominate heat transport.

**Fig. 4:**
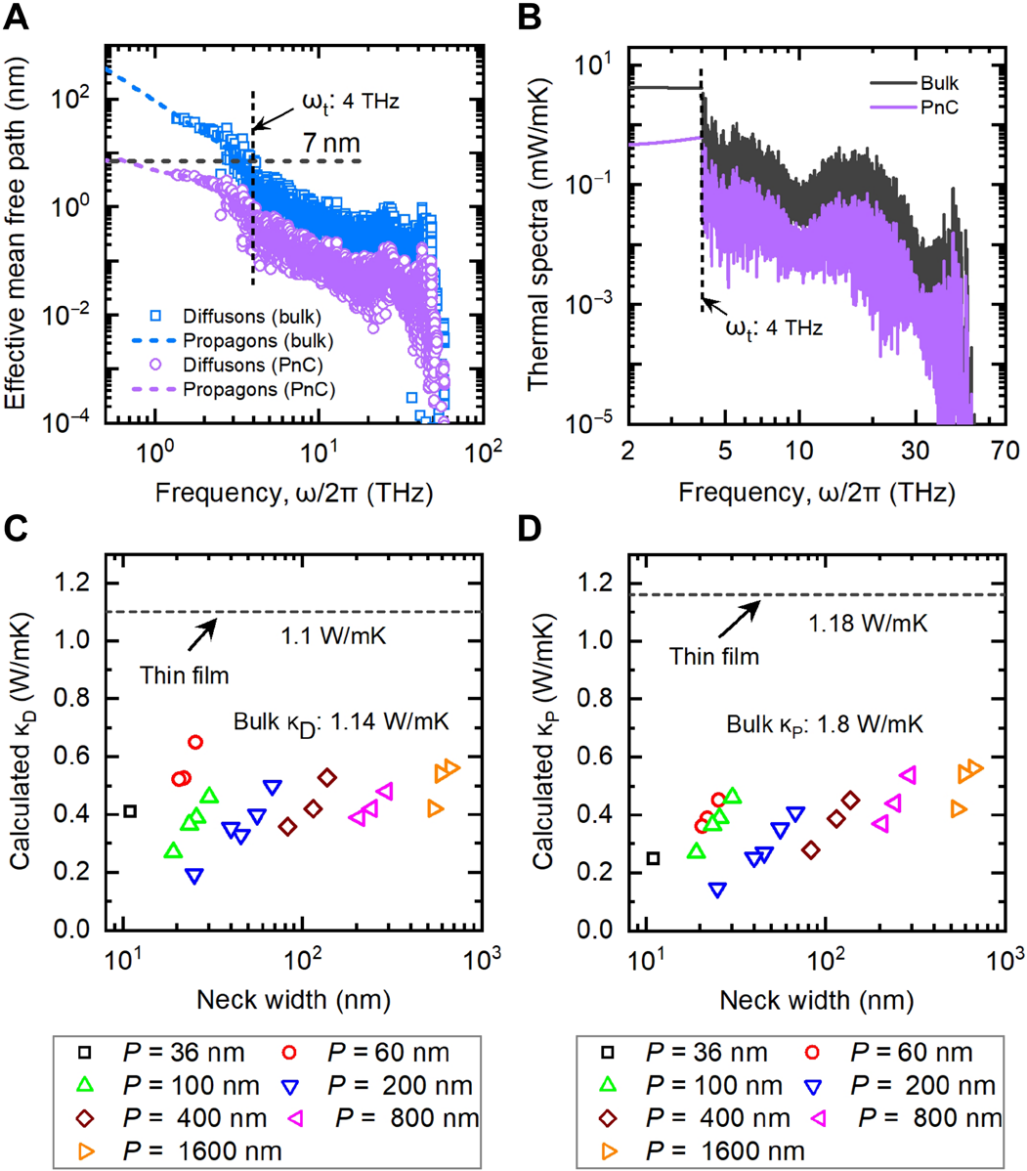
Calculated thermal properties of amorphous Si_3_N_4_. (**A**) Comparison of the effective MFP of propagons and diffusons for bulk a-Si_3_N_4_ and a-Si_3_N_4_ phononic materials, taking the sample with a pitch size *P* of 200 nm and a hole diameter *D* of 175 nm as an example. (**B**) Comparison of thermal conductivity κ spectra for bulk a-Si_3_N_4_ and a-Si_3_N_4_ phononic materials, taking the sample with *P* = 200 nm and *D* = 175 nm as an example. (**C**) and (**D**) are the calculated thermal conductivity contribution from diffusons (κ_D_) and propagons (κ_P_) for all samples as a function of neck width.

Now that we have demonstrated the validity of our model in amorphous PnCs, we investigate how the mode-dependent transport properties of propagons and diffusons are affected by the nanostructures. In Fig. 4A, we compare the MFPs of bulk a-Si_3_N_4_ and those of a-Si_3_N_4_ PnCs with *P* = 200 nm and *D* = 175 nm at 300 K. We see that the MFP of both propagons and diffusons is drastically suppressed in a-Si_3_N_4_ PnCs. For propagons in the PnCs, the MFPs are substantially reduced from that of the bulk case (10 to 100 nm) to sub–10 nm. Even for diffusons, the MFPs are reduced to about 16% of the bulk value, which is only a few angstroms. An important feature we noticed here is that the minimum MFP of propagons is around 10 nm. This indicates that propagon transport can be substantially disturbed by the PnC structures if we can reduce *n* to a few nanometers. The steep decrease in κ_mat_ below *n* of 20 nm shown in Fig. 2A can be understood well by the feature observed in Fig. 4A.

The shortened effective MFP leads to a substantial reduction in κ. Figure 4B shows a comparison of the frequency-dependent κ spectra between bulk a-Si_3_N_4_ and a-Si_3_N_4_ PnCs. The κ spectra of propagons and diffusons in PnCs are substantially reduced (nearly 90 and 80%, respectively) from those of bulk counterparts. As a result, κ_P_, κ_D_, and κ_T_ are reduced to 0.15, 0.19, and 0.34 W/mK, respectively, which is almost one order of magnitude smaller than κ_P_ (1.1 W/mK), κ_D_ (1.8 W/mK), and κ_T_ (2.9 W/mK) of the bulk case. The calculated κ_P_ and κ_D_ for all the a-Si_3_N_4_ PnCs are summarized in Fig. 4 (C and D), respectively. Because of the boundary scattering of propagons, the reduction in κ_P_ from the bulk value can exceed 70% even for samples with a large *n* of 665 nm. For samples with *n* below 20 nm, κ_P_ is smaller than 0.2 W/mK, which indicates that 90% of propagons are scattered. For diffusons, whose MFP varies from 0.5 to 10 nm in the bulk case, boundary scattering can still lead to a 40 to 80% reduction in κ_D_, depending on the *P* and *n*. This indicates that diffusive boundary scattering due to the PnC structure can reduce the transmittance of diffusons. These results suggest that PnC structures can not only effectively scattering propagons but also severely backscatter diffusons. The current findings not only reveal the mechanisms of thermal conductivity reduction in amorphous PnCs but also suggest that amorphous PnCs can realize the ultimate reduction of heat conduction of amorphous solids. Moreover, our analysis suggests that propagons and diffusons can be unified treated as quasiparticles with defined propagating or diffusive lengths, i.e., the MFPs, in the boundary scattering process, which indicates that in terms of boundary scatterings, there is no fundamental difference between propagons and diffusons.

## CONCLUSIONS

In summary, we have experimentally and theoretically demonstrated that nanostructured PnCs patterning can effectively modify the thermal transport properties of amorphous solids. The experimental results reveal that periodic cylindrical holes patterned on a-Si_3_N_4_ thin films can drastically decrease κ from the intrinsic bulk value, even realizing the amorphous diffusive limit when *n* is reduced below 20 nm. By defining the MFP of propagons and diffusions with a combination of the phonon kinetics gas model and AF theory, the measured κ of a-Si_3_N_4_ PnCs can be reproduced well without any fitting parameters. The analysis reveals that the MFP of both propagons and diffusons are substantially shortened by boundary scattering in ultrafine nanophononic structures, which results in substantial reductions in κ of amorphous solids. Moreover, the fact that the measured κ of a-Si_3_N_4_ PnCs was reproduced by the particle-based Boltzmann transport equation suggests that coherent transport is not substantial at 300 K even when *P* is reduced to 36 nm. The results demonstrate the possibility of applying the phonon engineering techniques developed in crystalline materials to amorphous materials, which opens a new door for manipulating thermal properties in microelectronic devices.

## MATERIALS AND METHODS

### Fabrication of PnCs

Amorphous PnCs are fabricated on a-Si_3_N_4_/Si/a-Si_3_N_4_ wafer by a semiconductor wafer process technology. The thickness of the Si layer was 500 μm. The low-stress a-Si_3_N_4_ layers were grown by LPCVD with a thickness of 70 nm. The samples were fabricated in a suspended bridge structure with a total length and width of 30 and 10 μm, respectively. Periodical through-holes at various pitch sizes ranging from 36 to 1600 nm were patterned on the a-Si_3_N_4_ layer. Electron beam lithography was used to fabricate samples at pitch sizes above 60 nm. On the other hand, directed self-assembly lithography was used to fabricate ultrafine 36-nm-pitch PnCs. After the hole patterning, suspended bridge structures were fabricated. The bridge shape on the top a-Si_3_N_4_ layer is patterned by a direct laser lithographer. A square-shaped Al pad with a dimension of 10 μm by 10 μm by 130 nm was then deposited on the bridge center. Last, the top a-Si_3_N_4_ layer was suspended by removing the Si under the bridge with anisotropic wet etching.

### Measurement of thermal conductivity

The TDTR method was used for κ measurement of the suspended a-Si_3_N_4_ PnCs. The transient response of heat dissipation through the amorphous PnCs was measured to obtain κ. A typical two-color pump-probe measurement was used for this measurement. Diode laser was used as the light source for both the pump beam and the probe beam. The pump beam and the probe beam were irradiated on the Al pad, and the temporal change of the reflected power of probe beam was monitored. Reflectivity *R* of the Al pad changes according to the temperature *T* change. The relation between *R* and *T* are described as, Δ*R* = α·*R*·Δ*T*, where Δ*R* and Δ*T* denote the change of reflectivity and temperature, and α is the thermal reflectance coefficient. We assume that α is constant within the heated temperature range. Thus, we could measure the *T*-dependent change of *R* of the Al pad. Since the suspended bridges are thermally isolated from the surrounding environment, the thermal energy generated on the heated Al pad dissipates through the suspended bridge. Therefore, the transient change in *R*, or namely, *T*, of the Al pad depends on the thermal properties of the suspended bridge. This enables us to determine κ of the suspended bridge structure based on this measurement. The TDTR signal decay is fitted by an exponential function with the following form

$$\text{signal}(t)=A_{1}\text{exp}[-\frac{\left(t-t_{0}\right)}{\tau_{1}}]+A_{2}\text{exp}[-\frac{\left(t-t_{0}\right)}{\tau_{2}}]+y_{0}$$

In addition, we carry out FEM analysis using the transient thermal analysis module of ANSYS software to obtain the relation between decay time τ_1_, τ_2_ and material thermal conductivity κ_mat_ for each phononic bridge geometry. By fitting the measured signal by the exponential curve, we obtain the best fit κ_mat_.

### Calculation of bulk thermal properties of silicon nitrides

The bulk a-Si_3_N_4_ structures were constructed by the typical melt-quenching method using MD with the Tersoff interatomic potentials (*36*) in the Large-scale atomic/molecular massively parallel simulator (LAMMPS) package (*37*), which was shown to work well for predicting the structures and thermal properties for amorphous (*8*). The parameters of Tersoff potential for a-Si_3_N_4_ are taken from Table 1 in the works of de Brito Mota *et al.* (*36*), which are obtained by fitting the atomistic forces from ab initio calculations. During the melt-quenching process, a 7560-atom crystal Si_3_N_4_ was first melted at 5000 K and then quenched to 300 K with a quenching rate of 0.05 K/ps in an isothermal-isobaric (NPT) ensemble. The structure was then relaxed at 300 K in an NPT and canonical (NVT) ensemble for 20 ns to reduce the residual stress and strain. Last, energy minimization was performed to obtain stable a-Si_3_N_4_ structures. The time steps in the MD calculations are set to 0.1 fs to cover the maximum frequency of the vibrational modes. The final geometry of the sample was a cuboid with a side length of 4.62, 3.98, and 4.41 nm, which was large enough to capture the character of the vibration modes in amorphous, as shown by the works of Larkin and McGaughey (*8*). The radius distribution function of the prepared a-Si_3_N_4_ sample clearly shows its amorphous structure character (fig. S2).

After the preparation of these amorphous structures, we are able to obtain the eigenmodes and frequencies of vibrational modes at the gamma point by using lattice dynamics in the General Utility Lattice Program (GULP) (*38*). The transition frequency of propagons and diffusons (ω_t_ = 4 THz) is obtained by identifying the ω^2^-dependent trend in DOS (fig. S3A) or the cutoff frequency of dynamic structure factor (effective dispersion relation) of propagons (fig. S3B), detailed information of which is summarized in section S2.

Vibrational mode relaxation time was obtained from the MD-based NMD method (*8*). The calculated frequency-dependent relaxation time and the diffusivity obtained from the AF theory, together with the diffusive velocity of diffusons, are shown in section S3.

## Supplementary Material

abc0075_SM.pdf

## SUPPLEMENTARY MATERIALS

Supplementary material for this article is available at http://advances.sciencemag.org/cgi/content/full/6/39/eabc0075/DC1
