# Supplementary material for: Ultimate suppression of thermal transport in amorphous silicon nitride by phononic nanostructure
Source: Sci Adv. 2020 Sep 25;6(39):eabc0075. doi: 10.1126/sciadv.abc0075 (PMC7518865; doi:10.1126/sciadv.abc0075)
Supplement: abc0075_SM.pdf [file abc0075_SM.pdf]

Science Advances
AAAS

advances.sciencemag.org/cgi/content/full/6/39/eabc0075/DC1

Supplementary Materials for

Ultimate suppression of thermal transport in amorphous silicon nitride by phononic nanostructure

Naoki Tambo*, Yuxuan Liao, Chun Zhou, Elizabeth Michiko Ashley, Kouhei Takahashi, Paul F. Nealey, Yasuyuki Naito, Junichiro Shiomi*

*Corresponding author. Email: tambo.naoki@jp.panasonic.com (N.T.); shiomi@photon.t.u-tokyo.ac.jp (J.S.)

Published 25 September 2020, *Sci. Adv.* **6**, eabc0075 (2020)
DOI: 10.1126/sciadv.abc0075

This PDF file includes:

Sections S1 to S3
Figs. S1 to S5
Table S1
References

Supplementary note 1: Accumulative thermal conductivity with propagon and phonon mean free path

Figure S1A shows the accumulative thermal conductivity κ of bulk amorphous Si_3N_4 (a-Si_3N_4) and bulk crystalline Si (c-Si) plotted as a function of propagon/diffuson mean free path (MFP) and phonon MFP, respectively. We also show the normalized accumulative κ of both materials in Fig. S1B. Note that the accumulative κ of a-Si_3N_4 and c-Si was obtained by our theoretical calculation explained in the main text. We see that most of the phonons that contribute to thermal transport are distributed in a MFP-range of 100 nm-10 μm in c-Si, whereas most of the propagons are distributed in a MFP-range of 1-100 nm. The difference in MFP distribution between the two materials gives rise to the different behavior in the κ vs. surface-to-volume (S/V) ratio curve described in the main text, where saturation of κ starts at a lower S/V ratio for c-Si PnCs than for a-Si_3N_4 PnCs.

Another noticeable feature in Fig. S1A and S1B is that we see a step-like feature around MFP of 6 nm in the accumulative-κ curve of a-Si_3N_4. As a result, propagons/diffusons with MFP of 1-6 nm contribute to only about 10% of the total κ. The step-like feature observed in Fig. S1A and S1A is consistent with the step like feature observed in Fig. 2A and 2B in the main text. The good agreement described here supports that saturation boundary scattering of particle-like propagons/diffusons are the main mechanism of κ reduction and saturation shown in Fig. 2A and 2B.

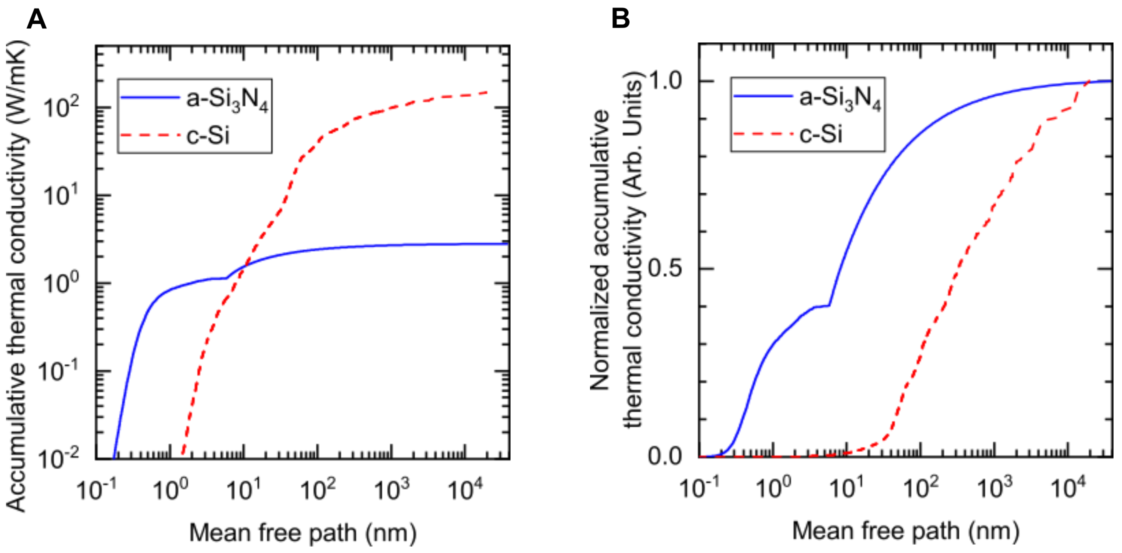

Fig.S1. Accumulative thermal conductivity of bulk solids. (**A**) Accumulative thermal conductivity and (**B**) normalized accumulative thermal conductivity of bulk amorphous Si_3N_4 (a-Si_3N_4) and crystalline Si (c-Si), plotted as a function of propagon/diffuson and phonon mean free path (MFP). The vertical axis in (A) is shown in a logarithmic scale. MFP of propagon/diffuson and phonon is used for a-Si_3N_4 and c-Si respectively.

Supplementary note 2: Radius distribution function and the transition frequency of propagons and diffusons in amorphous silicon nitride

The bulk a-Si_3N_4 structures were constructed by the typical melt-quenching method using molecular dynamics (MD) with the Tersoff interatomic potentials (*37*) in the LAMMPS packet (*38*), which was shown to work well for predicting the structures and thermal properties for amorphous (*8*). The parameters of Tersoff potential for a-Si_3N_4 are taken from Table 1 in the works of F. de Brito Mota *et al.* (*37*), which are obtained by fitting the atomistic forces from ab initio calculations. During the melt-quenching process, a 7560-atom crystal Si_3N_4 was first melted at 5000 K, and then quenched to 300 K with a quenching rate of 0.05 K/ps in an *NPT* ensemble. The structure was then relaxed at 300 K in an *NPT* and *NVT* ensemble for 20 ns to reduce the residual stress and strain. Finally, energy minimization was performed to obtain stable a-Si_3N_4 structures. The timesteps in the MD calculations are set to 0.1 fs to cover the maximum frequency of the vibrational modes. The final geometry of the sample was a cuboid with a side length of 4.62 nm, 3.98 nm and 4.41 nm, which was large enough to capture the character of the vibration modes in amorphous, as shown by the works of Larkin *et al*. (*8*). The radius distribution function of the prepared a-Si_3N_4 sample clearly shows its amorphous structure character (Fig. S2). The calculated densities of a-Si_3N_4 are 2800 kg/m^3, which matches well with the typical measured results.

The transition frequency between propagons and diffusions is obtained from the ω^2-dependent trend in vibrational mode density of states (*DOS*) (Fig. S3A) according to the works of Larkin *et al*. (8). The reason is that the effective dispersion relation (i.e. dynamic structure factor(8)) of propagons is linear (Fig. S3B), thus their *DOS* obeys the ω^2-dependent Debye approximation. Moreover, from Fig. S3B we also see that the effective dispersion relation of modes above 4 THz disappeared, which indicates vibrational modes above 4 THz in amorphous SiN are indeed diffusons. Note that since we also include boundary scattering of diffusons by defining their effective mean free path via the random walk theory, we do not need a very accurate definition of the transition frequency. A variation of the transition frequency does not affect the calculated total thermal conductivity of the amorphous phononic materials.

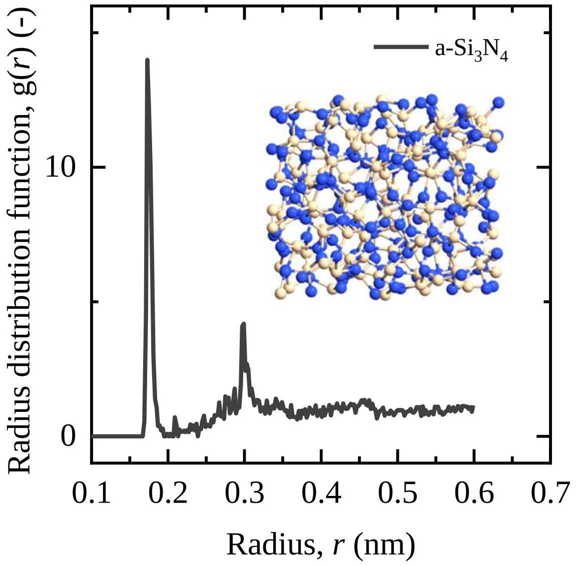

Fig. S2. Atomistic structures and radius distribution function of a-Si_3N_4.

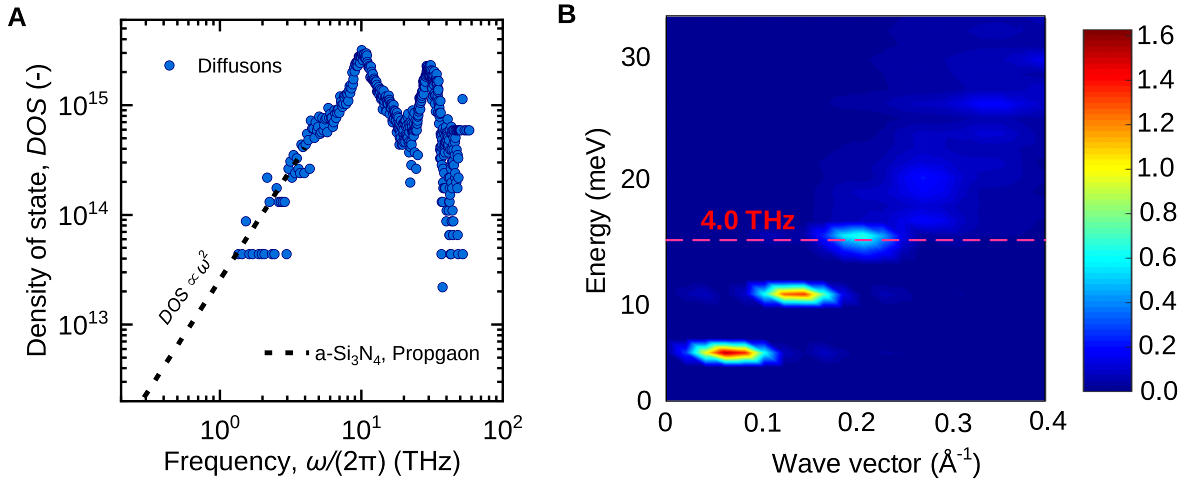

Fig. S3. Density of states and effective dispersion relations of the vibrational mode. The density of states (**A**) and effective dispersion relations (**B**) of the vibrational mode in a-Si_3N_4. We plotted the effective dispersion relation of the longitudinal branch as an example.

Supplementary note 3: Sound speed, frequency-dependent relaxation time, diffusivity and diffusive velocity of heat carriers in amorphous silicon nitride

Firstly, to valid our calculation of sound speed, we compared the calculated sound speed of longitudinal (S_L) and transversal modes (S_T) with experimental data (Table S1), which shows good agreement. After the validation, we obtained the appropriate sound speed v_s in Eq. 3 by fitting the *DOS* with the Debye model, as shown by the dashed line in Fig. S3A. A comparison of v_s and Debye sound speed (v_{dby}) is summarized in Table S1. An acceptable error of 16.7% was obtained. The reason for the difference can be that the viscosity damping lowers v_s of propagons, as revealed in the works of G. Baldi, *Rat* and Vacher (39–*41*).

Table S1. The predicted v_s, S_L, S_T for a-Si_3N_4, and a comparison with experimental measurements.

a-Si_3N_4	v_s (m/s)	S_L (m/s)	S_T (m/s)	v_{dby} (m/s)	(v_{bdy} - v_s)/ v_{bdy} (%)
This work	6200	12000	6680	7439	16.7%
Exp. Data (*32*)	-	10300	6200	6857	-

The calculation of the relaxation time for a-Si_3N_4 by NMD is similar to the works of Larkin *et al*. (*8*). Firstly, we reproduced the relaxation time of a-Si in the works of Larkin *et al*., which was further validated by experimental measurement using inelastic x-ray scattering (IXS) (39, *42*). We also did validation works for amorphous silica, which can well reproduce the experimental results via inelastic x-ray scattering (IXS) (39, *42*), picosecond optical technique (POT) (*43*), Brillouin ultraviolet scattering (BUVS) (*44*, *45*), and Brillouin light scattering (BLS) (*46*). The validation makes us confident for the calculation for the relaxation time of a-Si_3N_4. The results for a-Si and a-Si_3N_4 are shown in Fig. S4A and S4B, respectively. The diffusivity of propagons is obtained by Eq. (1) in the manuscript, while that for diffusons is from the AF theory (Fig. S5A). The diffusive velocity is calculated by Eq. (8) in the manuscript, the results along with v_s are summarized in Fig. S5B.

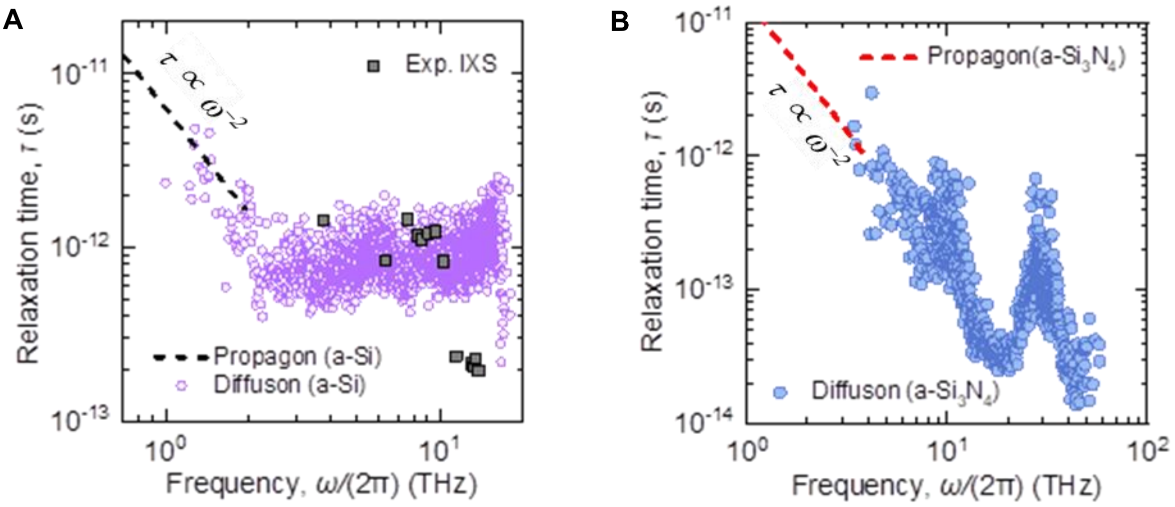

Fig. S4. Relaxation time of amorphous materials as a function of frequency: (**A**) a-Si; (**B**) a-Si_3N_4. The experimental data in (a) is obtained by IXS (39,*42*).

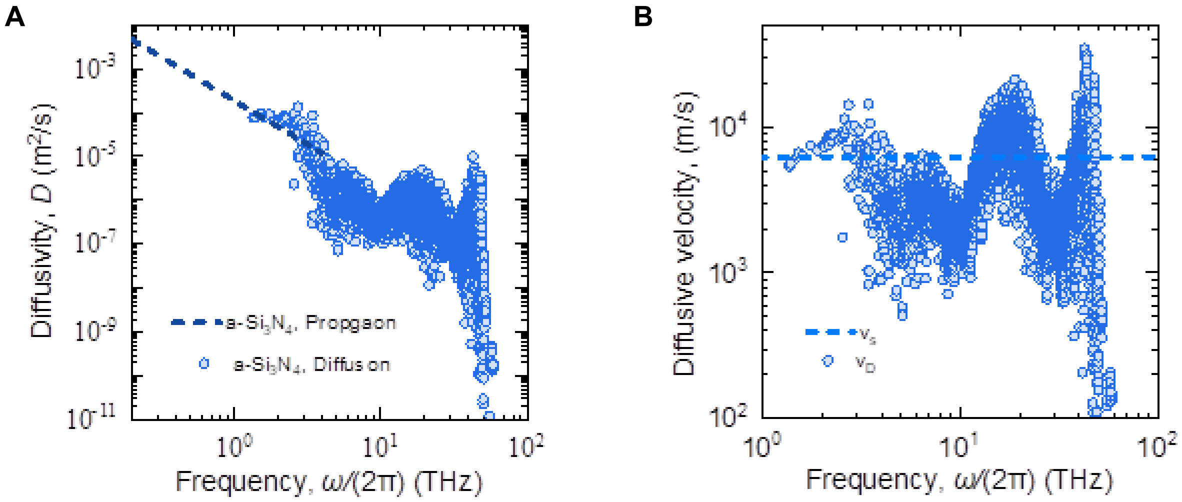

Fig. S5. Diffusivity and diffusive velocity for a-Si_3N_4. Diffusivity (**A**) and diffusive velocity (**B**) as a function of frequency for a-Si_3N_4.

REFERENCES

1. M. C. Wingert, J. Zheng, S. Kwon, R. Chen, Thermal transport in amorphous materials: A review. *Semicond. Sci. Technol.* **31**, 113003 (2016).

2. D. G. Cahill, W. K. Ford, K. E. Goodson, G. D. Mahan, A. Majumdar, H. J. Maris, R. Merlin, S. R. Phillpot, Nanoscale thermal transport. *J. Appl. Phys.* **93**, 793 (2003).

3. D. G. Cahill, P. V. Braun, G. Chen, D. R. Clarke, S. Fan, K. E. Goodson, P. Keblinski, W. P. King, G. D. Mahan, A. Majumdar, H. J. Maris, S. R. Phillpot, E. Pop, L. Shi, Nanoscale thermal transport. II. 2003-2012. *Appl. Phys. Rev.* **1**, 011305 (2014).

4. J. L. Feldman, M. D. Kluge, P. B. Allen, F. Wooten, Thermal conductivity and localization in glasses: Numerical study of a model of amorphous silicon. *Phys. Rev. B* **48**, 12589 (1993).

5. P. B. Allen, J. L. Feldman, Thermal conductivity of disordered harmonic solids. *Phys. Rev. B* **48**, 12581 (1993).

6. P. B. Allen, J. L. Feldman, J. Fabian, F. Wooten, Diffusons, locons and propagons: Character of atomic vibrations in amorphous Si. *Philos. Mag. B* **79**, 1715–1731 (1999).

7. F. DeAngelis, M. G. Muraleedharan, J. Moon, H. R. Seyf, A. J. Minnich, A. J. H. McGaughey, A. Henry, Thermal transport in disordered materials. *Nanoscale Microscale Thermophys. Eng.* **23**, 81–116 (2019).

8. J. M. Larkin, A. J. H. McGaughey, Thermal conductivity accumulation in amorphous silica and amorphous silicon. *Phys. Rev. B* **89**, 144303 (2014).

9. H. R. Seyf, A. Henry, A method for distinguishing between propagons, diffusions, and locons. *J. Appl. Phys.* **120**, 025101 (2016).

10. J. Moon, B. Latour, A. J. Minnich, Propagating elastic vibrations dominate thermal conduction in amorphous silicon. *Phys. Rev. B* **97**, 024201 (2018).

11. Y. Zhou, M. Hu, Record low thermal conductivity of polycrystalline Si nanowire: Breaking the Casimir limit by severe suppression of propagons. *Nano Lett.* **16**, 6178–6187 (2016).

12. T. Zhan, Y. Xu, M. Goto, Y. Tanaka, R. Kato, M. Sasaki, Y. Kagawa, Phonons with long mean free paths in a-Si and a-Ge. *Appl. Phys. Lett.* **104**, 071911 (2014).

13. J. L. Braun, C. H. Baker, A. Giri, M. Elahi, K. Artyushkova, T. E. Beechem, P. M. Norris, Z. C. Leseman, J. T. Gaskins, P. E. Hopkins, Size effects on the thermal conductivity of amorphous silicon thin films. *Phys. Rev. B* **93**, 140201(R) (2016).

14. R. Sultan, A. D. Avery, J. M. Underwood, S. J. Mason, D. Bassett, B. L. Zink, Heat transport by long mean free path vibrations in amorphous silicon nitride near room temperature. *Phys. Rev. B* **87**, 214305 (2013).

15. S. Kwon, J. Zheng, M. C. Wingert, S. Cui, R. Chen, Unusually high and anisotropic thermal conductivity in amorphous silicon nanostructures. *ACS Nano* **11**, 2470–2476 (2017).

16. L. Yang, Q. Zhang, Z. Cui, M. Gerboth, Y. Zhao, T. T. Xu, D. G. Walker, D. Li, Ballistic phonon penetration depth in amorphous silicon dioxide. *Nano Lett.* **17**, 7218–7225 (2017).

17. R. Chen, A. I. Hochbaum, P. Murphy, J. Moore, P. Yang, A. Majumdar, Thermal conductance of thin silicon nanowires. *Phys. Rev. Lett.* **101**, 105501 (2008).

18. A. I. Hochbaum, R. Chen, R. D. Delgado, W. Liang, E. C. Garnett, M. Najarian, A. Majumdar, P. Yang, Enhanced thermoelectric performance of rough silicon nanowires. *Nature* **451**, 163–167 (2008).

19. M. N. Luckyanova, J. Garg, K. Esfarjani, A. Jandl, M. T. Bulsara, A. J. Schmidt, A. J. Minnich, S. Chen, M. S. Dresselhaus, Z. Ren, E. A. Fitzgerald, G. Chen, Coherent phonon heat conduction in superlattices. *Science* **338**, 936–939 (2012).

20. J. Ravichandran, A. K. Yadav, R. Cheaito, P. B. Rossen, A. Soukiassian, S. J. Suresha, J. C. Duda, B. M. Foley, C.-H. Lee, Y. Zhu, A. W. Lichtenberger, J. E. Moore, D. A. Muller, D. G. Schlom, P. E. Hopkins, A. Majumdar, R. Ramesh, M. A. Zurbuchen, Crossover from incoherent to coherent phonon scattering in epitaxial oxide superlattices. *Nat. Mater.* **13**, 168–172 (2014).

21. J. Tang, H.-T. Wang, D. H. Lee, M. Fardy, Z. Huo, T. P. Russell, P. Yang, Holey silicon as an efficient thermoelectric material. *Nano Lett.* **10**, 4279–4283 (2010).

22. J.-K. Yu, S. Mitrovic, D. Tham, J. Varghese, J. R. Heath, Reduction of thermal conductivity in phononic nanomesh structures. *Nat. Nanotechnol.* **5**, 718–721 (2010).

23. M. Nomura, Y. Kage, J. Nakagawa, T. Hori, J. Maire, J. Shiomi, R. Anufriev, D. Moser, O. Paul, Impeded thermal transport in Si multiscale hierarchical architectures with phononic crystal nanostructures. *Phys. Rev. B* **91**, 205422 (2015).

24. M. Nomura, J. Shiomi, T. Shiga, R. Anufriev, Thermal phonon engineering by tailored nanostructures. *Jpn. J. Appl. Phys.* **57**, 080101 (2018).

25. T. Hori, J. Shiomi, C. Dames, Effective phonon mean free path in polycrystalline nanostructures. *Appl. Phys. Lett.* **106**, 171901 (2015).

26. C. Zhou, T. Segal-Peretz, M. E. Oruc, H. S. Suh, G. Wu, P. F. Nealey, Fabrication of nanoporous alumina ultrafiltration membrane with tunable pore size using block copolymer templates. *Adv. Funct. Mater.* **27**, 1701756 (2017).

27. C. Zhou, N. Tambo, E. M. Ashley, Y. Liao, J. Shiomi, K. Takahashi, G. S. W. Craig, P. F. Nealey, Enhanced reduction of thermal conductivity in amorphous silicon nitride-containing phononic crystals fabricated using directed self-assembly of block copolymers. *ACS Nano* **14**, 6980–6989 (2020).

28. H. Ftouni, C. Blanc, D. Tainoff, A. D. Fefferman, M. Defoort, K. J. Lulla, J. Richard, E. Collin, O. Bourgeois, Thermal conductivity of silicon nitride membranes is not sensitive to stress. *Phys. Rev. B* **92**, 125439 (2015).

29. M. Nomura, J. Nakagawa, Y. Kage, J. Maire, D. Moser, O. Paul, Thermal phonon transport in silicon nanowires and two-dimensional phononic crystal nanostructures. *Appl. Phys. Lett.* **106**, 143102 (2015).

30. J. Chen, G. Zhang, B. Li, A universal gauge for thermal conductivity of silicon nanowires with different cross sectional geometries. *J. Chem. Phys.* **135**, 204705 (2011).

31. R. Sultan, A. D. Avery, G. Stiehl, B. L. Zink, Thermal conductivity of micromachined low-stress silicon-nitride beams from 77 to 325 K. *J. Appl. Phys.* **105**, 043501 (2009).

32. B. L. Zink, F. Hellman, Specific heat and thermal conductivity of low-stress amorphous Si–N membranes. *Solid State Commun.* **129**, 199–204 (2004).

33. J. Yu, B. Sundqvist, B. Tonpheng, O. Andersson, Thermal conductivity of highly crystallized polyethylene. *Polymer* **55**, 195–200 (2014).

34. O. Koblinger, J. Mebert, E. Dittrich, S. Döttinger, W. Eisenmenger, P. V. Santos, L. Ley, Phonon stop bands in amorphous superlattices. *Phys. Rev. B* **35**, 9372(R) (1987).

35. J. Maire, R. Anufriev, R. Yanagisawa, A. Ramiere, S. Volz, M. Nomura, Heat conduction tuning by wave nature of phonons. *Sci. Adv.* **3**, e1700027 (2017).

36. F. de Brito Mota, J. F. Justo, A. Fazzio, Structural properties of amorphous silicon nitride. *Phys. Rev. B* **58**, 8323 (1998).

37. S. Plimpton, Fast parallel algorithms for short-range molecular dynamics. *J. Comput. Phys.* **117**, 1–19 (1995).

38. J. D. Gale, A. L. Rohl, The General Utility Lattice Program (GULP). *Mol. Simul.* **29**, 291–341 (2003).

39. G. Baldi, V. M. Giordano, G. Monaco, B. Ruta, Sound attenuation at terahertz frequencies and the boson peak of vitreous silica. *Phys. Rev. Lett.* **104**, 195501 (2010).

40. E. Rat, M. Foret, G. Massiera, R. Vialla, M. Arai, R. Vacher, E. Courtens, Anharmonic versus relaxational sound damping in glasses. I. Brillouin scattering from densified silica. *Phys. Rev. B* **72**, 214204 (2005).

41. R. Vacher, E. Courtens, M. Foret, Anharmonic versus relaxational sound damping in glasses. II. Vitreous silica. *Phys. Rev. B* **72**, 214205 (2005).

42. J. Moon, R. P. Hermann, M. E. Manley, A. Alatas, A. H. Said, A. J. Minnich, Thermal acoustic excitations with atomic-scale wavelengths in amorphous silicon. *Phys. Rev. Mater.* **3**, 065601 (2019).

43. A. Devos, M. Foret, S. Ayrinhac, P. Emery, B. Rufflé, Hypersound damping in vitreous silica measured by picosecond acoustics. *Phys. Rev. B* **77**, 100201(R) (2008).

44. C. Masciovecchio, G. Baldi, S. Caponi, L. Comez, S. di Fonzo, D. Fioretto, A. Fontana, A. Gessini, S. C. Santucci, F. Sette, G. Viliani, P. Vilmercati, G. Ruocco, Evidence for a crossover in the frequency dependence of the acoustic attenuation in vitreous silica. *Phys. Rev. Lett.* **97**, 035501 (2006).

45. P. Benassi, S. Caponi, R. Eramo, A. Fontana, A. Giugni, M. Nardone, M. Sampoli, G. Viliani, Sound attenuation in a unexplored frequency region: Brillouin ultraviolet light scattering measurements in $v{-}\mathrm{Si\,O_2}$. *Phys. Rev. B* **71**, 172201 (2005).

46. R. Vacher, J. Pelous, Behavior of thermal phonons in amorphous media from 4 to 300 K. *Phys. Rev. B* **14**, 823 (1976).
